# Histotripsy Ablation in Preclinical Animal Models of Cancer and Spontaneous Tumors in Veterinary Patients: A Review

**DOI:** 10.1109/TUFFC.2021.3110083

**Published:** 2021-12-31

**Authors:** Alissa Hendricks-Wenger, Lauren Arnold, Jessica Gannon, Alex Simon, Neha Singh, Hannah Sheppard, Margaret A. Nagai-Singer, Khan Mohammad Imran, Kiho Lee, Sherrie Clark-Deener, Christopher Byron, Michael R. Edwards, Martha M. Larson, John H. Rossmeisl, Sheryl L. Coutermarsh-Ott, Kristin Eden, Nikolaos Dervisis, Shawna Klahn, Joanne Tuohy, Irving C. Allen, Eli Vlaisavljevich

**Affiliations:** 1 Graduate Program in Translational Biology, Medicine and Health, Virginia Polytechnic Institute and State University, Roanoke, VA, 24016; 2 Department of Biomedical Engineering and Mechanics, Virginia Polytechnic Institute and State University, Blacksburg, VA 24061; 3 Department of Mechanical Engineering, Virginia Polytechnic Institute and State University, Blacksburg, VA 24061; 4 Department of Biomedical Sciences and Pathobiology, Virginia-Maryland College of Veterinary Medicine, Blacksburg, VA 24061; 5 Department of Basic Science Education, Virginia Tech Carilion School of Medicine, Roanoke, VA 24016; 6 Division of Animal Sciences, University of Missouri, Columbia, MO 65211; 7 Department of Large Animal Clinical Sciences, Virginia-Maryland College of Veterinary Medicine, Blacksburg, VA 24061; 8 Department of Small Animal Clinical Sciences, Virginia-Maryland College of Veterinary Medicine, Blacksburg, VA 24061; 9 ICTAS Center for Engineered Health, Virginia Tech, Kelly Hall, Blacksburg, VA 24061; 10 Department of Internal Medicine, Virginia Tech Carilion School of Medicine, Roanoke, VA 24016

**Keywords:** Therapeutics, Medical Transducers, Medical Imaging, System & Device Design

## Abstract

New therapeutic strategies are direly needed in the fight against cancer. Over the last decade, several tumor ablation strategies have emerged as stand-alone or combination therapies. Histotripsy is the first completely non-invasive, non-thermal, and non-ionizing tumor ablation method. Histotripsy can produce consistent and rapid ablations, even near critical structures. Additional benefits include real-time image-guidance, high precision, and the ability to treat tumors of any predetermined size and shape. Unfortunately, the lack of clinically and physiologically relevant pre-clinical cancer models is often a significant limitation with all focal tumor ablation strategies. The majority of studies testing histotripsy for cancer treatment have focused on small animal models, which have been critical in moving this field forward and will continue to be essential for providing mechanistic insight. While these small animal models have notable translational value, there are significant limitations in terms of scale and anatomical relevance. To address these limitations, a diverse range of large animal models and spontaneous tumor studies in veterinary patients have emerged to complement existing rodent models. These models and veterinary patients are excellent at providing realistic avenues for developing and testing histotripsy devices and techniques designed for future use in human patients. Here, we provide a review of animal models used in preclinical histotripsy studies and compare histotripsy ablation in these models using a series of original case reports across a broad spectrum of preclinical animal models and spontaneous tumors in veterinary patients.

## I. INTRODUCTION

Histotripsy is a focused ultrasound ablation method that destroys tissue through the precise control of acoustic cavitation [1–4]. Using microsecond long, high-pressure pulses applied by a focused ultrasound transducer coupled to the patient, a histotripsy bubble cloud is generated non-invasively inside the targeted tissue [5–9]. The rapid expansion and collapse of these bubbles result in complete disruption of the target tissue into acellular debris [10]. As a non-thermal ablation method, histotripsy has shown the potential to overcome the limitations associated with thermal ablation. For instance, histotripsy has been shown to produce consistent and complete ablation in the liver, which is highly vascular, when applied through abdominal or transcostal acoustic windows [1, 3, 11, 12]. Histotripsy has also shown the ability to ablate tissue near vital structures such as major vessels, bile ducts, and nerves while preserving these structures [1, 3, 9, 13, 14]. Due to these features, histotripsy is currently being developed for multiple clinical applications, most notably for the treatment of cancer [1, 3, 4, 15]. However, despite the promise of histotripsy and the many positive results reported in preclinical studies, to date, only a single human clinical trial has been conducted to test histotripsy for the treatment of cancer [16]. This recent Phase I clinical trial was conducted with non-curative, palliative intent in patients with multifocal primary and metastatic liver tumors, with results showing histotripsy could safely target and ablate targeted liver tumors [16]. To our knowledge, no other human clinical trials of histotripsy for the treatment of cancer have been conducted, limiting the potential widespread translation of histotripsy into clinical practice as an improved non-invasive tumor ablation method.

Similar to other focal tumor ablation modalities, one of the primary factors limiting the rapid translation of histotripsy into the clinic has been the lack of appropriate animal models for studying the safety, effectiveness, and optimization of histotripsy for the treatment of different cancers. Previous efforts to develop histotripsy for specific oncological applications have been split into separate studies testing human-scale histotripsy systems for targeting and ablating healthy tissue in large animal models that have similar anatomy and physiology to human patients (primarily pigs) [1, 3, 11, 12]. These studies are typically complemented by separately testing histotripsy tumor ablation in rodents (mice, rats) using customized small animal devices that allow histotripsy to be applied in this setting, albeit not using the systems or acoustic parameters that will ultimately be developed for use in humans [2, 4, 17, 18]. In this paper, we provide a review of animal models used in preclinical histotripsy studies and compare histotripsy ablation in these models using a series of original case reports from a broad spectrum of preclinical animal models and spontaneous tumors in veterinary patients.

### A. Murine Models for Histotripsy Development

Murine models are the dominant *in vivo* model in the biomedical engineering and cancer biology fields and are essential in acquiring fundamental data. Mice are easy to house and handle, relatively inexpensive, share many genetic similarities to humans, and there are many transgenic and knock-out lines available to study specific disease mechanisms, proteins, and pathways [19–25]. In typical mouse models used to study tumor ablation modalities, most studies are mouse or human cell line based [4, 17, 26–33]. While these models offer excellent reproducibility and standardization, they rarely recapitulate the clinical, *in situ*, microenvironment found in patients [32, 34]. This is especially true in subcutaneous, flank generated tumors. Orthotopic tumors can improve insight but still suffer from cell line specific limitations related to cancer biology. Spontaneous and induced tumors in small animals are more physiologically relevant to human patients [15, 35–37]; however, the inability to predict tumor development, location, progression, and general lack of reproducibility are all limitations to these models. Likewise, the lack of translational acoustic windows, limited imaging, lack of precision, and lack of physiological relevance are all major limitations of small animal models in the development of histotripsy and other focused ultrasound modalities. While critical for proof-of-concept testing and mechanistic insight [4, 17, 27–29], the miniaturization of the systems has significantly slowed the rate of clinical translation for imaging, targeting, and effectively treating different tumors types. As a result, there is a significant need for improved preclinical large animal tumor models for studying histotripsy tumor ablation using clinically relevant devices and treatment parameters to enable the more rapid and successful translation of histotripsy into clinical practice for the treatment of cancer in humans.

### B. Porcine Models for Histotripsy Development

Large animal models can address many of the shortcomings often cited for rodents, in many cases offering direct translation from animal to human. For example, the same tumor ablation systems can typically be utilized, similar clinical/surgical techniques can be refined, and many large animal models accurately recapitulate human anatomical and physiological features. This is critical for imaging and ultrasound-based modalities such as histotripsy. However, the increased size and complexity of the species results in several significant limitations typically associated with cost, the requirement for specialized housing, and the need for unique institutional resources. Most large animal models are based on spontaneous tumor development, which has some of the same limitations commonly cited for small animal studies. Recent technological advancements in gene editing technologies have largely negated these issues, specifically in pig models. In general, pigs have yet to play a significant role in experimental oncology [38]. However, with the rise of technologies capable of inducing genetic modifications in large mammals, such as CRISPRs and TALONs, there is a renewed interest in the pig as a cancer model. This is especially true for medical devices, where the utilization of the same devices, instruments, and clinical practice can be utilized in pigs and humans, enabling a more rapid translation to clinical trials. Over the last decade, genetically modified porcine cancer models have been generated targeting APC, TP53, KRAS, GLI2, V-H-RAS, and BRACA1[38]. Indeed, the recently developed “Oncopig” cancer model (OCM) has the potential to bring the pig to the forefront of tumor models [39]. These novel transgenic swine models recapitulate human cancer through Cre recombinase induced site and cell-type specific expression of KRAS and TP53 transgenes [40]. To express Cre, pigs must be either crossed with transgenic animals expressing cell type specific Cre or direct injection into the tissue type of interest with an adenoviral vector encoding Cre recombinase [39]. Both KRAS and TP53 mutations are common in multiple cancers and multiple OCM models appear to be currently under development [39].

The OCM model is expected to have several strengths, including an accurate recapitulation of the tumor microenvironment, accurate immune system profile, and the outbred animals are expected to better model human patients [39]. However, the reliance on Cre recombinase and associated delivery vectors will result in an unpredictable number of tumors that form in random locations. Likewise, while tumor development in the OCM model is rapid, it is highly difficult to predict tumor progression in this model. This can add significant cost and time. As an alternative strategy, research teams have focused on immunocompromised pig models, where deletions in the *RAG2* and *IL2RG* genes have generated animals with similar characteristics to NOD scid gamma (NSG) mice, which are susceptible to human tissue engraftment [41]. Similar to NSG mice, human tumor cell lines, and patient-derived xenograft (PDX) tissue can be engrafted. This allows a highly predictable tumor that can be surgically placed subcutaneously or orthotopically in a human-sized animal with similar anatomy and physiology. Tumors can be expanded to increase tissue availability for *ex vivo* studies or surgically placed near critical structural elements (such as blood vessels or nerve bundles) to evaluate and model tumor ablation in hard-to-treat locations within the organ. Another advantage of this strategy is the use of human cells in a pig model. This allows robust identification, evaluation, and quantification of the ablation zone and metastatic tumors at the histopathologic level using human antibodies in porcine tissues. The lack of a functional immune system is a clear disadvantage of immunocompromised pig models, and they require housing under highly specialized germfree conditions that are only available at a limited number of institutions.

### C. Veterinary Clinical Oncology Patient Populations for Histotripsy Development

Beyond tumor models, spontaneous cancers in pet animals offer unique translational research opportunities for cancer imaging and device development that provide a robust combination of clinical and physiological relevance to humans. Indeed, comparative oncology studies have revealed significant similarities between many canine and human cancers [42, 43]. Companion animals, like humans, are genetically diverse and are exposed to many of the same environmental influences as their owners. Canine cancer patients have been used in numerous studies to provide translatable pharmacokinetic and pharmacodynamic data [44–49], and these trials have been critical for determining the scheduling/dosing of small molecular inhibitors in humans [50]. The spontaneous nature of canine cancers, their biologic similarities to human cancers, and the anatomic similarities between dogs and people offer highly relevant clinical conditions for device development.

In the veterinary clinic, soft tissue tumors are currently the most accessible tumor types to treat using histotripsy and the data from subcutaneous tumors in animal models can be directly applied. Tumors of the skin and subcutaneous tissues are common in dogs, with malignant tumors comprising 20–40% of skin masses submitted for histopathology [51]. Soft tissue sarcomas (STS) arise from the connective tissues, comprising 15% of all skin tumors in dogs, and are subclassified pathologically based on the tissue of origin [52]. Common tumor types seen in clinical practice include fibrosarcoma, perivascular wall tumor, peripheral nerve sheath tumor, liposarcoma, and myxosarcoma. Histologic distinction can be challenging and may be of minor clinical importance as the majority of STS share a similar biological behavior and follow a similar course of treatment: the patient could be cured with wide surgical excision, rarely requiring adjuvant chemotherapy. The anatomical distribution varies, with most STS occurring on the trunk and limbs. Generally, STS are slow-growing and not life-threatening. However, in veterinary practice, this often results in medical attention being sought late in the course of disease when the curative surgical treatment option may be limb amputation, a disfiguring surgery, or a curative surgery is no longer possible. The range of tumor sizes seen in typical veterinary specialty practice can range from a few centimeters up to 20 cm.

**Fig. 1.**
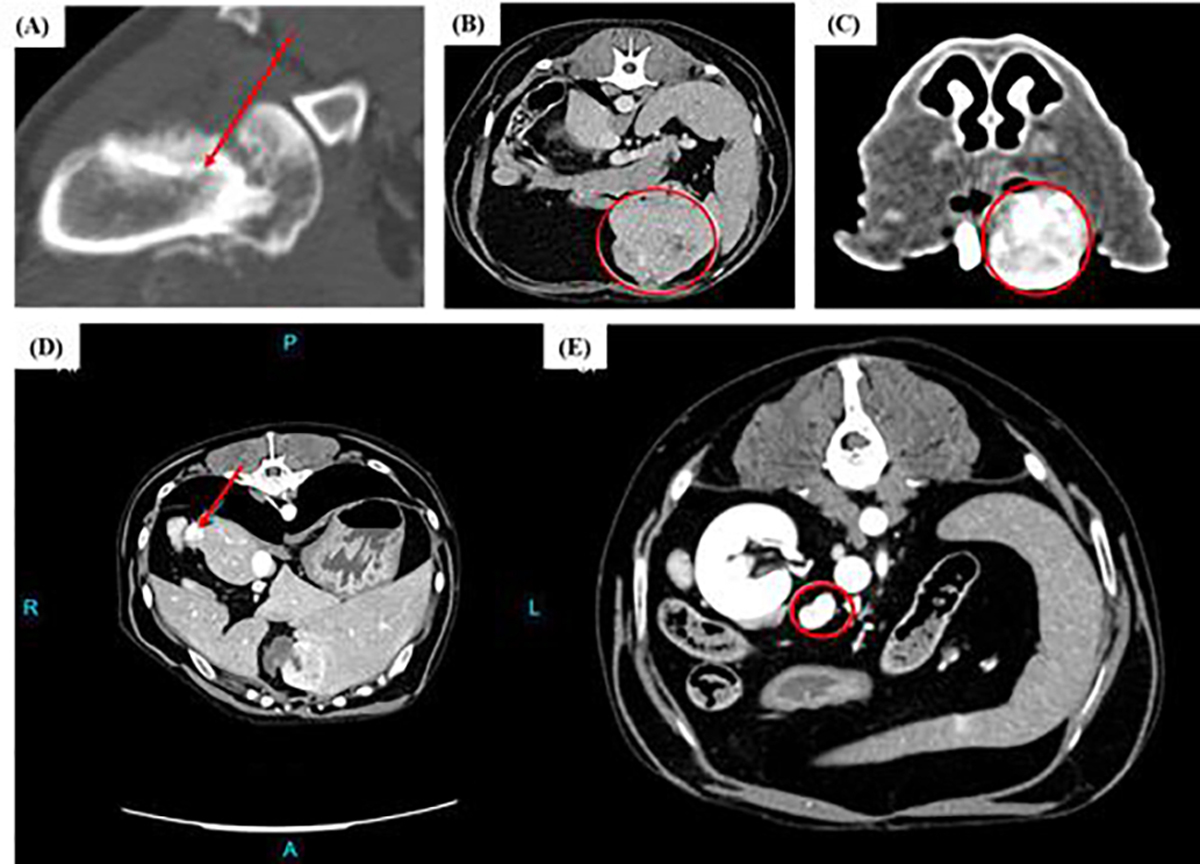
Example Spontaneous Tumors in Canines. (A) CT image of a rostral maxillary tumor in a dog, indicated in circled area. Note the aggressive invasion of the tumor into the maxilla, causing bone lysis. (B) CT image of a humeral osteosarcoma in a dog, indicated by the arrow. Note the aggressive nature of the tumor, causing bone lysis and proliferation. (C) CT image of a liver tumor in a dog, indicated by the circle. (D) CT image of a pancreatic tumor in a dog, indicated by the arrow. (E) CT image of a metastatic lymph node in the same patient, indicated by a circle.

Osteosarcoma (OS) is a primary bone cancer and a devastating disease for both human and canine patients. It is the most common primary bone tumor in children and adolescents, as well as, in the dog [53, 54]. Figure 1A shows a CT image of an osteosarcoma in the forelimb of a dog. Treatment of OS involves removal of the primary tumor, and chemotherapy to inhibit metastatic disease. The main cause of death in canine and human patients with OS remains metastatic disease. Advancements in OS therapy need to focus on treating both primary and metastatic tumors. Dogs and humans with OS share striking similarities that make the dog an exceptional comparative oncology research model. These similarities include matching biological behavior, nearly identical histological features, shared global gene expression signatures, and similar responses to standard treatment regimens [55–59]. For example, most OS lesions in dogs and humans occur in the appendicular skeleton around the metaphyseal region of long bones, and the disease tends to be associated with large and/or tall individuals [54, 55]. The anatomic similarities between the canine and human skeletal structures allow the dog to be an accurate model for histotripsy systems development and treatment evaluation studies. Osteosarcoma appears radiographically identical in humans and dogs, displaying a lesion on CT and MRI scans with a combination of lytic and proliferative bone [55, 60]. These radiographic similarities allow for cross-species comparisons when evaluating histotripsy treatment outcomes using imaging modalities. The strength of naturally occurring canine OS as a model for comparative oncology histotripsy research lies not only in the similarities of the disease in both species but also in non-disease-related factors. The dog is an outbred species with an intact immune system that develops spontaneously occurring OS over an extended period, like humans and unlike inbred laboratory rodents. Dogs with OS reflect the heterogeneity of human patients with OS, displaying interindividual variations in tumor characteristics. Both humans and dogs with OS share similar clinical disease manifestations and outcomes. The >10-fold increased incidence of canine OS (estimated incidence of 13.9/100,000) compared to human OS (1.02/100,000), the shorter lifespan of dogs, the rapid progression of metastatic OS in dogs enable outcomes to be assessed in relatively short periods compared to their human counterparts [55, 61, 62]. Metastatic bone tumors also occur in dogs, and common primary cancers that metastasize to bone include mammary carcinoma, prostatic carcinoma, urogenital carcinoma. Osteosarcoma can also metastasize to bone. Histotripsy, with its ability to disintegrate solid tumors and its potential for immunogenic stimulation, is uniquely poised to have the potential to achieve the goals of OS therapy – treating both the primary tumor and metastatic lesions.

Liver cancer is a preferred target for histotripsy and is the focus of the first human clinical trial of the ablation strategy in patients [16]. Primary liver cancer in humans is the fifth most common cause of cancer death in men and the ninth most common cause of cancer death in women. Over the past two decades, incidence rates have increased, and the overall mortality rates have been rising an average of 2.6 percent each year [63]. At present, only 10–23% of human patients with HCC are surgical candidates for curative-intent treatment [64, 65]. Liver transplantation is the only therapeutic option that offers a consistent 90% survival rate, but the scarcity of liver donors severely limits its broad applicability [66]. Primary liver tumors in dogs are considered infrequent and account for 1.5% of all canine tumors, with hepatocellular carcinoma (HCC) being the most common (Fig. 1B) [67]. The prognosis is good for massive HCC when surgical resection is possible [68]. In contrast, the prognosis is poor for dogs with malignant tumors other than massive HCC, and especially for dogs with nodular and diffuse HCC, as surgical resection is not possible. Literature suggests that 40% of canine HCC are of nodular or diffuse presentation and that a significant proportion of the massive HCC tumors are non-resectable with complete margins due to their hilar or bile duct association. Bland embolization and chemoembolization have been reported with variable success in the palliation of four dogs with HCC [69].

Canine oral tumors are another tumor type that can serve as strong comparative oncology research models for their human counterparts. For instance, canine oral melanoma has been shown to represent a faithful model for human mucosal melanoma and human triple wild-type melanoma [70–72]. The disease in both species shares genetic signatures, clinical behavior, and histological characteristics [73, 74]. Head and neck squamous cell carcinomas are the most common oral cancer in people, and canine oral squamous cell carcinoma shares similarities with the human form of this disease [75]. Patients with oral cancers commonly present with clinical signs associated with pain due to the oral cancer, decreased appetite, and weight loss. Oral tumors in dogs occur with relatively low frequency, constituting 6% of all canine cancers, and three of the most common oral cancers in dogs are malignant melanoma, squamous cell carcinoma, and fibrosarcoma [76–78]. Oral osteosarcoma (OS) represents 12 – 13% of all canine OS [79]. Canine oral tumors are commonly locally invasive and many are aggressively metastatic, especially to the locoregional lymph nodes. A non-surgical tumor ablation technique such as histotripsy, which is also capable of stimulating an anti-tumor immune response and can be used for tissue selective ablation of tumors near critical structures [1, 80–82], has exciting potential to benefit both canine and human patients suffering from oral cancers. Figure 1C shows an example of a canine oral tumor that is locally invasive, causing bone lysis of the rostral maxilla.

Similar to humans, pancreatic cancer in dogs is also a critically important malignancy. Pancreatic tumors in dogs can originate from the exocrine pancreas, for example, adenocarcinomas of the pancreatic ducts or acini, or originate from the endocrine pancreas, for example, insulinoma. Insulinoma is the most common pancreatic endocrine tumor in dogs, originates from pancreatic β cells and secretes insulin, causing potentially life-threatening hypoglycemia [83]. Insulinoma also aggressively metastasizes to other organs, commonly to the liver and regional lymph nodes. Figure 1D–E shows CT images of a pancreatic tumor with its associated lymph node metastasis in a dog. Canine insulinomas behave in a biologically similar fashion to human malignant insulinoma [84]. The functional insulin-secreting nature of primary and metastatic insulinoma is a major life-limiting factor for patients, and treatment of *both* primary and metastatic disease is essential for improving prognosis. Surgical resection of all gross disease in dogs with pancreatic cancer, though not curative, is recommended to improve the efficacy of medical adjuvant treatments and disease outcomes [83]. Complete surgical resection of all gross disease is not always achievable, depending on the location and extent of the lesions. Resection of tumors located in the body of the pancreas is associated with high morbidity and potentially mortality rates, and the extent of metastatic lesions may preclude surgical resection. Novel treatment approaches are needed to improve the long-term survival of pancreatic cancer patients. New treatment techniques that can non-surgically ablate tumor lesions are attractive alternates to surgical tumor resection. Treatment approaches to stimulate the host immune system to mount an anti-tumor immune response that carries immune memory will improve the prognosis for the development or recurrence of metastatic disease in pancreatic cancers. Histotripsy, with its ability to precisely ablate solid tumors and its potential for immunogenic stimulation, is uniquely poised to advance the therapeutic efficacy and prognosis of insulinomas, a tumor with currently limited treatment options.

Despite significant progress over the last decade, brain tumors such as malignant gliomas (MG), represent some of the most treatment-refractory cancers in both humans and dogs, and local treatment failures remain a significant source of morbidity and mortality in brain tumor patients. Dogs and humans are the only mammals known to spontaneously and commonly develop MG, and MG accounts for approximately 35–40% of all primary brain tumors in dogs. As dogs have a complex gyrencephalic brain, and canine gliomas share many clinical, imaging, histomorphologic, molecular, and genetic features with human gliomas, dogs with brain tumors are uniquely suited translational model systems for the study of tumor ablation strategies [85]. In both dogs and humans, there is a dire need for new approaches that can be applied to tumors involving eloquent brain regions, circumvent or disrupt the blood brain barrier, target highly resistant tumors and cancer stem cells, generate minimal collateral damage to healthy tissue, and promote an anti-tumor immune response. Successful therapeutic strategies are likely to require a combination approach involving surgery, chemo and radiation therapy, and novel ablation approaches that can de-bulk the primary tumor and attenuate recurrence driven from residual cancer stem cells repopulating the resected tissue, or predictably and reversibly disrupt the blood brain barrier in a penumbra of tissue surrounding the bulk tumor to facilitate the delivery of therapeutics that are normally impermeant to the brain [86]. Unlike many of the other tumors discussed thus far, a significant challenge to the application of histotripsy in the brain is the thickness inhomogeneities inherent to the skull, which can cause aberrations in transcranially propagated ultrasound pulses resulting in reduced focal pressure amplitudes in the target tissue. Thus, a craniectomy is required to provide an acoustic window for effective bubble cloud formation with our systems. To overcome current obstacles associated with histotripsy in the brain, our research team is planning to utilize a recently developed acoustically transparent cranial implant [87] and is rapidly adapting clinical histotripsy systems for use in active clinical trials in dogs with brain tumors.

### D. Objectives of Case Reports

In the sections below, we provide a series of case reports from established small animal tumor models, as well as, emerging large animal models, under development by our group, for studying histotripsy tumor ablation. First, we show representative results of histotripsy tumor ablation in small animal (murine) cancer models including examples of subcutaneous, orthotopic, and patient-derived xenograft (PDX) models. Next, we show results from a recently developed immunocompromised *RAG2/IL2RG* deficient pig tumor model that better mimics human anatomy and physiology compared to mouse models. Finally, we discuss the opportunity to investigate the safety and efficacy of histotripsy in veterinary populations of dogs with spontaneous tumors by providing examples of our preliminary studies of histotripsy in these veterinary patients. Overall, these studies provide an overview of the benefits and limitations of currently available animal models, emerging large animal tumor models, and veterinary cancer patients to enable more rapid and reliable clinical translation of histotripsy for the treatment of cancer patients.

## II. PROCEDURES

### A. Histotripsy Systems and Pressure Calibrations

**Fig. 2.**
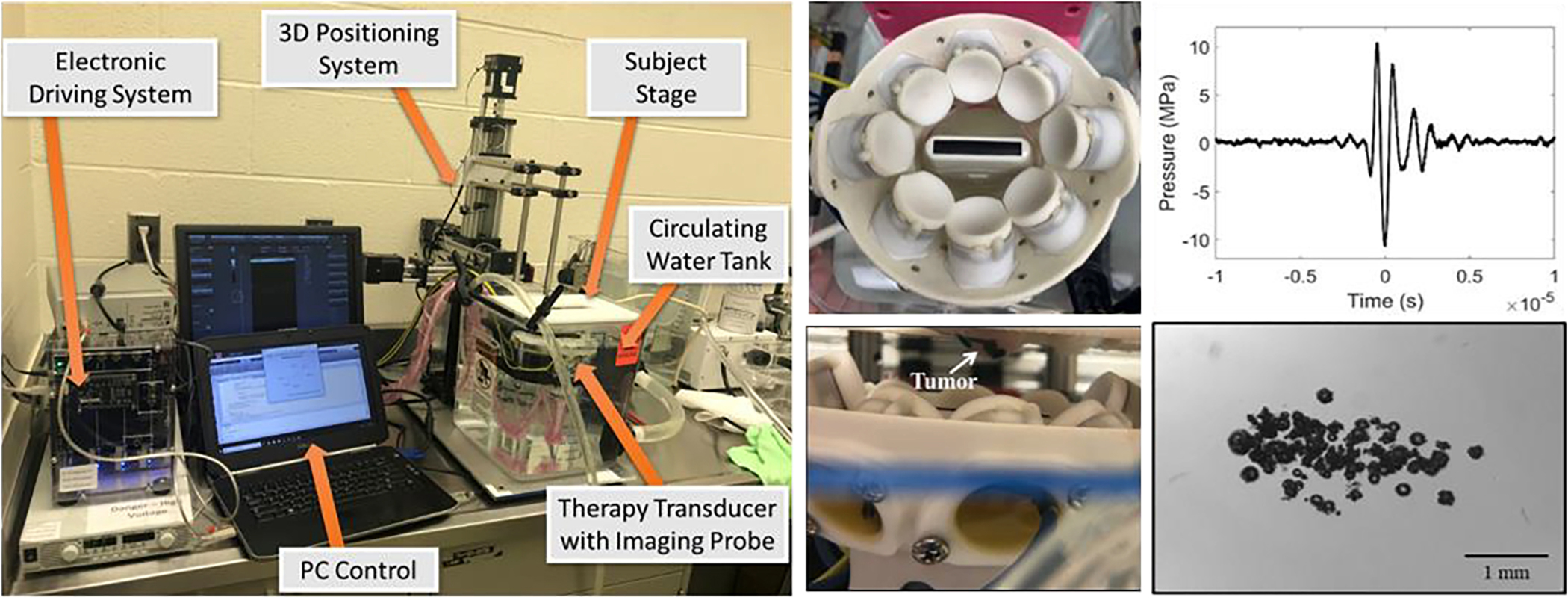
Example Small Animal Histotripsy System. (A) A small animal system consisting of a 1) 1 MHz therapy transducer with an 2) imaging transducer coaxially aligned and mounted to a 3) 3D motorized positioning system to deliver histotripsy to target submerged in degassed water on a 5) subject stage. A custom-built electronic driving system is controlled with computer software. (B) Linear ultrasound imaging probe coaxially aligned with 1 MHz therapy transducer used to (C) treat tumors in vivo above the cavitation threshold (>24 MPa). (D) Acoustic waveform for 1 MHz transducer collected with a hydrophone at a peak negative pressure of ~10 MPa. (E) 1 MHz bubble cloud captured on a high-speed camera at a treatment-relevant peak negative pressure of ~32 MPa.

Two separate histotripsy systems were used in this study for the small animal and large animal *in vivo* histotripsy studies. The small animal *in vivo* experiments were conducted using a custom 8-element 1MHz histotripsy transducer (Fig. 2B). The design features of the transducer are summarized in Table 1. The transducer was driven via a custom high-voltage pulser designed to generate short therapy pulses of <2 cycles controlled by a field-programmable gate array (FPGA) board (Altera DE0-Nano Terasic Technology, Dover, DE, USA) programmed for histotripsy therapy pulsing. The transducer was positioned in a tank of degassed water beneath a custom-designed mouse surgical stage and attached to a computer-guided 3-D positioning system with 0.05 mm motor resolution to control the automated volumetric treatments (Fig. 2A). A linear ultrasound imaging probe with a frequency range of 10–18 MHz (L18–10L30H-4, Telemed, Lithuania, EU) was coaxially aligned inside the transducer for treatment guidance and monitoring (Fig. 2C). The transducer was powered by a high voltage DC power supply (GENH750W, TDK-Lambda), and the system was controlled using a custom user-interface operated through MATLAB (MathWorks). This design has been used previously for multiple small animal histotripsy studies in mice and rats [2, 4, 17, 18].

**Fig. 3.**
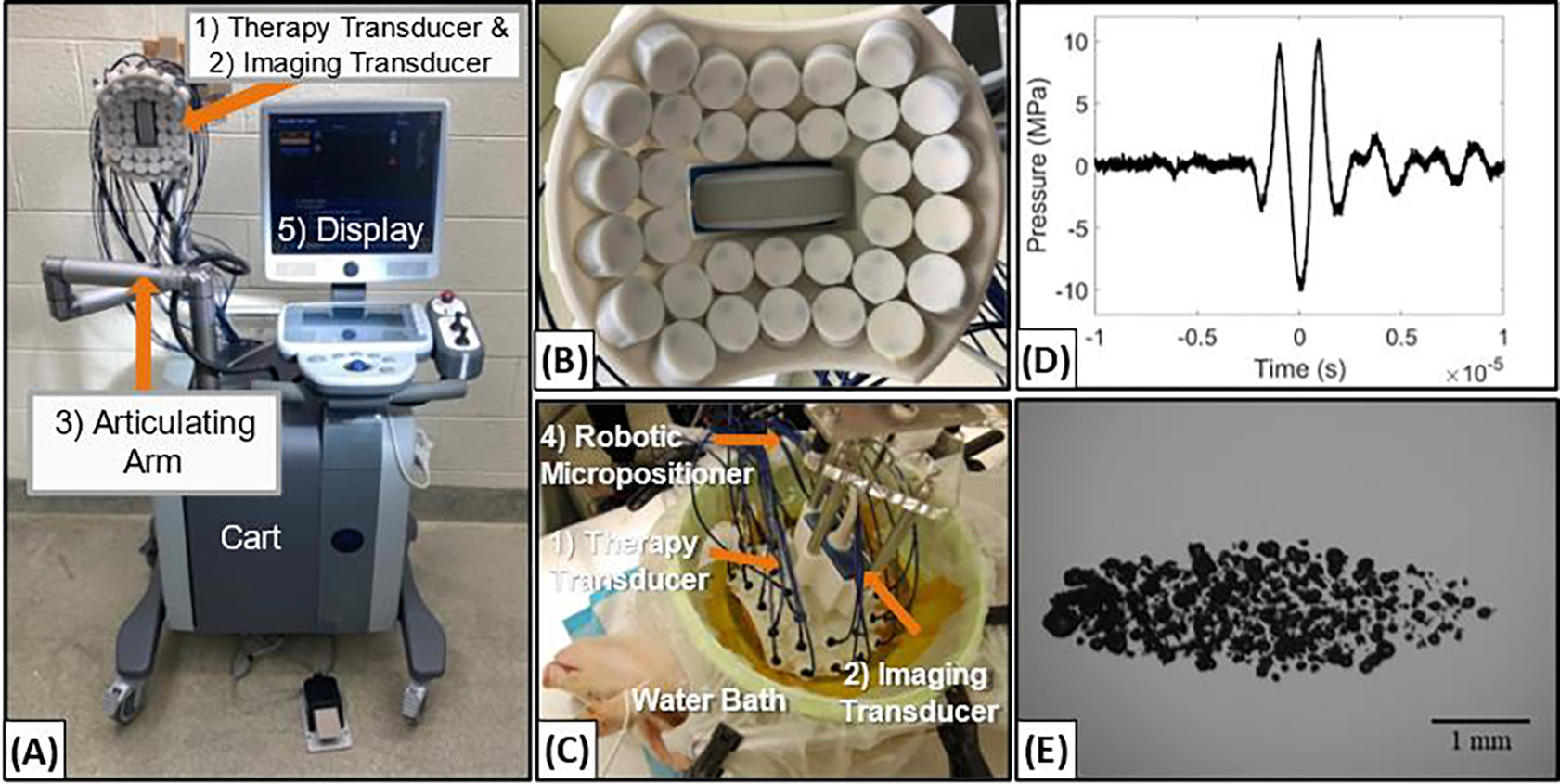
Example Large Animal Histotripsy System. (A) Histotripsy system designed for liver cancer ablation consisting of 1) therapy and 2) imaging transducers attached to a 3) articulating arm with a 4) motorized micro-positioner controlled by 5) custom software. (B) Image of therapy transducer with co-axially aligned imaging probe used for (C) previous in vivo liver studies. (D) Acoustic waveform for 500 kHz transducer collected with a hydrophone at a peak negative pressure of ~10 MPa. (E) 500 kHz bubble captured on a high-speed camera at a treatment-relevant peak negative pressure of ~33 MPa.

For the large animal *in vivo* experiments, histotripsy was applied using a custom 32-element 500 kHz array transducer with design features summarized in Table 1 (Fig. 3B). The transducer was again driven via custom high-voltage pulser and FPGA board designed to generate short therapy pulses of <2 cycles, as described above. The transducer was fixed onto a prototype clinical histotripsy treatment cart (HistoSonics, Ann Arbor, MI, USA) that includes an ultrasound imaging system, robotic micro-positioner, and customized software for applying volumetric histotripsy ablations (Fig. 3A). A 3 MHz curvilinear imaging probe (Model C5–2, Analogic Corp., Peabody, MA) was coaxially aligned through a central hole in the therapy transducer in order to allow real-time treatment guidance and monitoring (Fig. 3B). The transducer was triggered from the HistoSonics system, powered by the same high voltage DC power supply used for the small animal system, and mounted onto the robotic micro-positioner in order to allow histotripsy to be uniformly applied to a predetermined volume within the tissue, as outlined in a previous study [1]. This overall system is similar to one used in prior large animal histotripsy studies conducted on healthy pigs as well as a recent Phase I clinical trial of histotripsy for treating liver cancer patients [1, 16], with the only difference being the custom 32-element transducer used in this study in place of the clinical histotripsy transducer used in the prior work.

### B. Hydrophone Focal Pressure Measurements, Beam Profiles, and Optical Imaging

Focal pressure waveforms for the 1MHz and 500kHz transducers were measured by a high sensitivity rod hydrophone (HNR-0500, Onda Corporation, Sunnyvale, CA, USA) and a custom-built fiber optic probe hydrophone (FOPH) [88, 89] in degassed water at the focal point of each transducer. The rod hydrophone was used to measure the 1D focal beam profiles of the transducer in the lateral, elevational, and axial directions at a peak negative pressure (*p*−) of ~1.8 MPa. Full width half-maximum dimensions at the geometric focus of each transducer were measured and recorded in Table 1. Focal pressures were directly measured with the FOPH up to a *p*− of ~20 MPa. At *p*− greater than ~20 MPa, *p*− values could not be measured directly because cavitation was stabilized at the tip of the FOPH fiber. At higher pressure levels (*p*− > 20 MPa), the focal pressure was measured by summing measurements from subsets of a quarter and half of the total elements on each transducer [42]. All waveforms were measured using a Tektronix TBS2000 series oscilloscope at a sample rate of 500MS/s, with the waveforms averaged over 128 pulses and recorded in MATLAB.

The bubble clouds generated by each transducer were characterized using high-speed optical imaging as described in previous studies [90, 91]. Briefly, the focal region inside 1% agarose tissue phantoms was imaged after acoustic propagation for each pulse using the 1 MHz and 500 kHz therapy transducers described above. Optical imaging was performed using a high-speed camera (FLIR Blackfly S monochrome, BFS-U3–32S4M-C 3.2 MP, 118 FPS, Sony IMX252, Mono, FLIR Integrated Imaging Solutions, Richmond, BC, Canada) and a 100 mm F2.8 Macro lens (Tokina AT-X Pro, Kenko Tokina Co., LTD, Tokyo, Japan). The camera was externally triggered to record one image per applied pulse, and the tissue phantom was backlit by a custom-built pulsed white-light LED strobe light capable of high-speed triggering with 1 μs exposures. Strobe duration was kept low (3–5 μs) and all exposures were centered at a delay of 8.5 μs after pulse arrival at the focus. This timing schema was previously demonstrated as the optimum delay for visualizing complete bubble clouds [90, 91]. The bubble cloud dimensions observed on optical imaging were subsequently used to determine the spacing between adjacent treatment points in *in vivo* tumor ablation experiments. Measured bubble cloud sizes for each transducer differed depending on the pressure applied during bubble cloud generation. For instance, the bubble cloud dimensions at a *p*− of 33 MPa in the axial, transverse, and elevational directions were 2.5 mm, 1.25 mm, and 1.25 mm and 5.0 mm, 1.5 mm, and 1.5 mm for the 1 MHz and 500 kHz systems, respectively. Example high speed images of the bubble cloud generated by the 1 MHz and 500 kHz transducer are shown in Figure 2E and Figure 3E, respectively.

### C. Murine Tumor Models

The ability of histotripsy to ablate tumors in *in vivo* mouse models was tested using the 1 MHz small animal histotripsy system to target tumors in three murine cancer models including 1) a subcutaneous renal cell carcinoma (RCC) model, 2) a subcutaneous cholangiocarcimona patient-derived xenograft (PDX) model, and 3) a 4T1 orthotopic mammary tumor model. In the subcutaneous RCC model, male BALB/cCr mice (n=4) were injected in the right flank with 6×10^6^ Renca RCC cells (ATCC CRL-2947) in Matrigel. In the subcutaneous cholangiocarcinoma (CC) PDX model, female Nod scid gamma (NSG) mice (n=4) with subcutaneous flank PDX CC tumors were commercially acquired from the Jackson Laboratory (PDX Specimen TM01225), through a process previously described [32]. In the orthotopic mammary tumor model, female BALB/c mice (n=4) were injected with 1.2 X 10^6^ murine 4T1 mammary tumor cells (ATCC CRL-2539) in the abdominal mammary glands. Once tumors were palpable, animals were monitored three times weekly and tumor diameter was determined by taking two perpendicular measurements, one of which was the widest point of the tumor. A single tumor diameter was calculated as the square root of the product of the two measurements. The mice underwent histotripsy treatment when their tumors reached the targeted treatment size ~0.5–1.0 cm in diameter.

For all tumors, histotripsy was applied once each tumor reached the predetermined target size. For all treatments, mice were anesthetized with inhaled isoflurane and secured to a platform over a tank of degassed water, with the temperature maintained between 35–39°C. Before treatment, fur was removed over the tumor and the surrounding area by applying Nair (Naircare, Ewing, NJ, USA) for 1–2 minutes and removed by wiping off with a wet paper towel. For treatment, mice were anesthetized with isoflurane (1.5 L/min oxygen flow rate with 1.0–2.5% isoflurane) and placed prone on the subject stage with their tumor submerged underwater (Fig. 2A). Animals’ respiratory rate and rhythm were monitored during treatment by trained personnel. Histotripsy was applied to a predetermined volume of the tumor using the custom 1MHz small animal transducer described above and shown in Figure 2. During treatment, histotripsy was applied using single cycle pulses applied at a pulse repetition frequency (PRF) of 250 Hz with a 1 second dwell time at each treatment point. To apply a volumetric treatment, the focus was raster-scanned through a series of treatment points spaced by 1.5 mm in the axial direction and 0.5 in the lateral for the Renca and 4T1 tumors. Given that the CC tumors are stiffer and more resistant to histotripsy, a PRF of 500 Hz with a 1 second dwell time was used to double the per point dose and the spacing was reduced to 0.75 mm and 0.25 mm to increase the overlap between adjacent treatment points. All study procedures complied with the NIH Guide for the Care and Use of Laboratory Animals and with the Virginia Tech Institutional Animal Care and Use Committee.

### D. RAG2/IL2RG Deficient Porcine Tumor Model

An ideal animal model for studying histotripsy tumor ablation using clinical prototype systems would 1) be implantable so that specific locations could be chosen for the hypothesis being tested, 2) allow implantation of human tumor cell lines to closely mimic the natural history and composition of human tumors, and 3) be in an animal large enough to evaluate human-scale devices. To accomplish these goals, our team has developed a series of immunodeficient pigs including *RAG2/IL2RG* double knockout pigs using CRISPR/Cas9 [92–98]. The methodology for generating the CRISPR/Cas9 has been previously reported [99, 100]. Briefly, target regions were PCR amplified using Dream Taq DNA Polymerase (Thermo Fisher Scientific). PCR conditions were as follows: initial denature at 95 °C for 2 min; denature at 95 °C for 30 sec, annealing at 60 °C for 30 sec, and extension at 72 °C for 30 sec for 34 cycles; 72 °C for 5 min; and holding at 4 °C. The PCR amplicons were sequenced to determine the mutation types generated by the CRISPR/Cas9 system. Immunocompromised state was verified with flow cytometry to analyze the presence of B (CD79A), T (CD3E), and NK (CD3E−/CD56+) cells.

Pigs were delivered through a sterile hysterectomy process previously described by a board-certified large animal veterinarian [101]. Pigs were monitored regularly until fully recovered from anesthesia and able to eat independently. Tail snips were taken from each piglet while still under the influence of anesthesia for genotyping, and ears were clipped for numbering. The pigs were born into sterile, gnotobiotic isolators and were fed sterile boxed milk throughout the study to prevent infections [101].

Human Panc01 cells (National Cancer Institute DTP, DCTP Tumor Repository) were propagated in DMEM supplemented with 10% FBS and 1% penicillin/ streptomycin and removed from plates with Trypsin in EDTA. While the pigs were still under the effect of anesthesia from the hysterectomy, 1.2×10^6^ of the following human tumor cell lines were injected: Panc01 (pancreas); U-251 (brain); HT-29 (colon); A549 (lung); or HepG2 (liver). Tumor cells were injected, subcutaneously, in 100 μL of Matrigel behind the right ear, with an additional 100 μL of Matrigel behind the left ear. Tumors were measured three times per week with plastic Vernier calipers. The diameter was calculated as the square root of the product of the widest diameter and the diameter perpendicular to that. At the same point of tumor measurement, weight and other health monitoring was also completed. In addition to the subcutaneous cell line-based tumors, we have also conducted orthotopic engraftment with human PDX tissues and organoids [41, 97], significantly expanding the utility of these model animals. All study procedures complied with the NIH Guide for the Care and Use of Laboratory Animals and with the Virginia Tech Institutional Animal Care and Use Committee.

### E. Histotripsy Treatment of Porcine Pancreas and Liver

The ability to xenograft tumor cell lines and PDX tissue in the *RAG2/IL2RG* knockout pigs orthotopically, provides a highly unique model for histotripsy development. Our initial studies have focused on the pancreas, due in part to the high relevance and difficulty in targeting that includes similar acoustic windows to that expected in human patients. Initial studies have focused on refining the imaging and targeting healthy organs with histotripsy in preparation for planned tumor ablation studies in the *RAG2/IL2RG* knockout orthotopic pig model. Animals were anesthetized with Telazol-Ketamine-Xylazine (TKX) and maintained under anesthesia for the duration of the imaging and histotripsy treatment. Pigs were cleaned, dried, and had the area of interest shaved and Nair (Naircare, Ewing, NJ, USA) was applied for 10 minutes before being washed with water to thoroughly remove hair that could interfere with imaging. Before treatment, freehand ultrasound imaging was used to identify the desired treatment location using both a linear ultrasound imaging probe with a frequency range of 10–18 MHz (L18–10L30H-4, Telemed, Lithuania, EU) and a 3 MHz curvilinear imaging probe (Model C5–2, Analogic Corp., Peabody, MA). For treatment, the histotripsy transducer and coaxial imaging probe were mounted onto a mechanical arm with an associated micropositioning system to provide a uniformed volumetric ablation guided by treatment planning software (Histosonics Inc., Ann Arbor, MI) [1]. The focus of the transducer was aligned on the imaging screen before treatment by generating a bubble cloud in degassed water. For treatment, the transducer was grossly positioned over the pig’s abdomen and the focus was aligned to the targeted location using ultrasound imaging. The transducer was coupled to the pig using a degassed water bath and an adhesive drape, as shown in Figure 3C. Once this location was identified, the transducer was turned on and histotripsy was applied to the target using single cycle pulses and a PRF of 500Hz, with the pressure slowly increased until a bubble cloud was generated in the tissue. The transducer was scanned to cover a 1 cm diameter spherical ablation volume with 500 pulses applied per treatment location. Real-time ultrasound imaging was used to monitor the treatment throughout the procedure. After treatment, freehand ultrasound imaging was again conducted to assess for any tissue damage, and the animals were then immediately euthanized per protocol by an intracardiac injection of pentobarbital sodium and phenytoin sodium (Euthasol). A full necropsy was conducted post-treatment by a board-certified veterinary pathologist (S.C.O. or K. E.). All study procedures complied with the NIH Guide for the Care and Use of Laboratory Animals and with the Virginia Tech Institutional Animal Care and Use Committee.

### F. Spontaneous Tumors in Canine Patients

In addition to the development of the SCID-like pig tumor model described above, our team has recently initiated three studies testing histotripsy tumor ablation in spontaneous tumors in client-owned dogs at the Animal Cancer & Research Center (ACCRC), Virginia-Maryland College of Veterinary Medicine (VMCVM). These prospective clinical trials evaluate the safety and feasibility of treating canine soft tissue sarcoma and osteosarcoma tumors, which are currently underway, as well as a study for treating canine brain tumors and others in future studies. These initial trials parallel previous veterinary clinical trials at our site treating canine soft tissue sarcoma, liver, brain, pancreatic, and lung cancers with either irreversible electroporation (IRE) or High Intensity Focused Ultrasound (HIFU) thermal ablation. All of these canine tumor populations represent potential candidates for future histotripsy studies. All clinical trials are approved by the Institutional Animal Care and Use Committee and the Veterinary Hospital Board. Client-owned dogs with naturally occurring tumors are recruited for these clinical trials through the ACCRC referral network and by registry of the trial on a publicly accessible, national veterinary clinical trials database [102]. All dogs undergo a screening process to determine eligibility for trial enrollment, including a physical examination, mass cytology or histopathology, complete blood count (CBC), serum biochemistry profile, and thoracic and abdominal imaging. Standard-of-care treatment options are always presented to owners and dogs are only included in studies if they meet the inclusion/exclusion criteria and if a written owner’s informed consent is obtained. Before a study treatment is administered for our current histotripsy clinical trials, enrolled dogs will typically undergo cross-sectional imaging of the tumor either with contrast-enhanced CT or MRI. These imaging studies enable visualization of the tumor characteristics as well as the surrounding and overlying tissues to assess the available acoustic window for each study. The planned histotripsy treatment is patient-specific and scheduled as appropriate, with all further diagnostic evaluation and treatments performed by the Histotripsy Clinical Team at the ACCRC.

### G. Histotripsy Ablation of Spontaneous Canine Tumors

The feasibility of histotripsy tumor ablation in canine patients with spontaneously-occurring tumors was tested in two dogs, one with a peripheral soft tissue sarcoma and one with a hindlimb osteosarcoma tumor, using the 500 kHz clinic prototype histotripsy system (Fig. 3). Patient-specific histotripsy treatment plans were designed prior to each treatment based on pre-treatment CT imaging, physical examinations, and ultrasound imaging assessments. These measures were used to determine the acoustic window and the prescribed area within the tumor that was targeted with histotripsy. For treatment, the patients were placed under general anesthesia following standard anesthetic protocols for client-owned patients. Anesthesia was maintained with inhaled isoflurane. The fur was shaved as closely to the skin as possible over the planned treatment site. Histotripsy was applied to 3cm diameter spherical volumes within the tumor by sweeping the focal point across the target volume using the robotic micro-positioner directed by the planning software. Vital signs (heart rate, blood pressure, temperature, ECG, and SpO_2_) were monitored throughout the procedure to assess patient safety and identify any potential physiologic disturbances. To ensure acoustic propagation from the transducer to the skin, a water bolus was coupled to the canine patient over the optimal acoustic window identified using pre-treatment CT images and freehand ultrasound imaging prior to treatment. The tumor was treated with histotripsy under ultrasound guidance using single cycle pulses applied at a PRF of 500 Hz. Before starting the volumetric treatment, the pressure at the focus was increased until a bubble cloud was generated at the focus. A volumetric ablation volume was then generated using the automated treatment strategy guided by the micro-positioner and software of the clinical histotripsy system. During histotripsy, the bubble cloud and tissue effects were monitored using real-time US imaging. Upon completion of the treatment, patients were recovered from anesthesia and were discharged into the care of their owners. The patients received a physical examination and evaluation of the skin overlying the histotripsy treatment site one day after treatment. Grading of adverse events was determined according to the Veterinary Cooperative Oncology Group-Common Terminology Criteria for Adverse Events (VCOG-CTCAE). A post-treatment CT scan with contrast was performed one day after treatment to assess the completeness of the ablation zone, the contrast enhancement pattern in the ablation zone, and the integrity of any critical structures within or adjacent to the ablation zone. Surgical resection was performed one day (osteosarcoma) or four days (soft tissue sarcoma) after histotripsy treatment. A second post-treatment CT scan was performed immediately before resection for the soft tissue sarcoma group. The patients were placed under general anesthesia following standard anesthetic protocols for client-owned animals to facilitate standard surgical resection of their tumors. The histotripsy-treated area of tumor and bone (for osteosarcoma patients), and all relevant adjacent and overlying tissue were inspected for gross signs of damage and harvested for histological evaluation. All study procedures complied with the NIH Guide for the Care and Use of Laboratory Animals.

### H. Histology & Morphological Analysis

After treatment, tissue specimens were visually inspected and fixed in 10% formalin for at least 24 hours before sectioning and staining. All tissues were stained using hematoxylin and eosin (H&E) to assess for histotripsy damage and any other changes in structure and density of collagen and other tissue structures (i.e. vessels and ducts) within the ablation volume. To determine the estimated extent of tissue ablated within the targeted volume, individuals trained by a board-certified veterinary pathologist compared regions of cellular damage with regions that were outside of the ablation zone. Results were then verified by a veterinary pathologist (S.C.O.) to ensure accuracy and avoid biases.

## III. RESULTS

### A. Histotripsy Treatment of Subcutaneous Murine Tumors

**Fig. 4.**
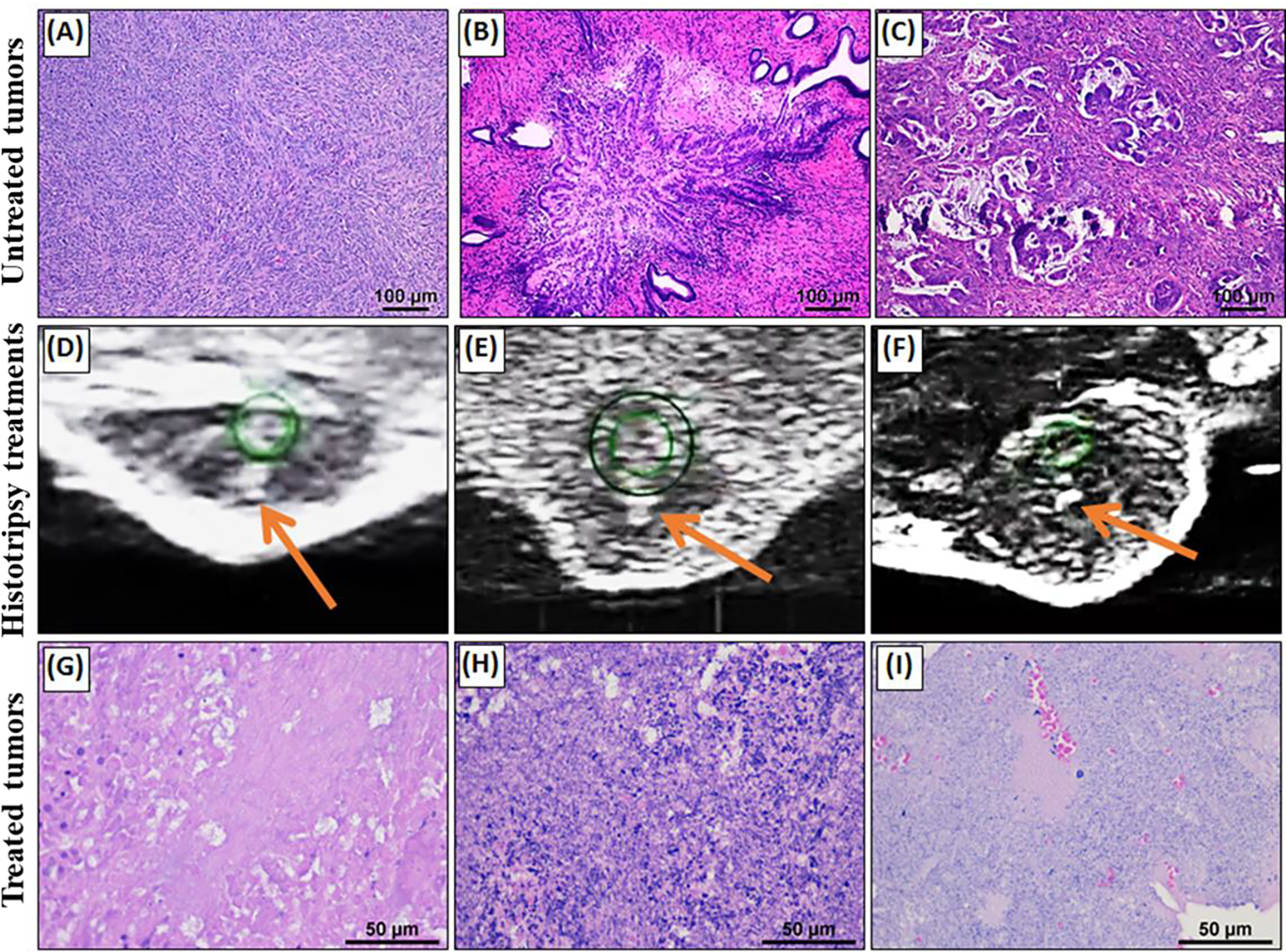
Histotripsy Tumor Ablation in Murine Models. (A) Subcutaneous murine pancreatic Pan02 tumor. (B) Representative primary human pancreatic tumor. (C) Human CC PDX murine xenografted tumor. (D) Ultrasound images of histotripsy bubble clouds forming in RCC, (E) 4T1, and (F) CC PDX tumors. (G-I) Histology demonstrating ablation within histotripsy targeted (G) RCC, (H) 4T1, and (I) CC PDX tumors.

Although a wide variety of subcutaneous and orthotopic tumor models have been established in mice, the majority of histotripsy studies, including the example histotripsy treatments shown in this study have focused on subcutaneous tumor injection models that allow straightforward assessments of tumor progression and treatment with minimal concerns regarding acoustic windows. In general, nodular tumors developed at the injection site for all cell line and PDX based tumors. When studying cell line-based tumors in mice, it is well characterized that most tumors tend to be less physiologically similar to the respective spontaneous tumors in human patients. For example, the pancreatic Pan02 tumors are densely cellular with only minimal fibrovascular stroma (Fig. 4A). The tumors often have variably-sized foci of necrosis, which typically develop centrally within the tumor. In terms of morphology, the tumor cells typically are arranged in vague, indistinct streams. The cells often exhibit significant pleomorphism, including differences in nuclear size and shape across the population with numerous mitotic figures observed throughout cells within the tumor. This is significantly different compared to a representative primary pancreatic tumor from human patients (Fig. 4B). We have found human patient derived xenograft (PDX) tumors in NOD scid gamma (NSG) mice to be highly useful in reproducing the complexity and stroma of the human primary tumor, including for studies of pancreatic cancer [32]. The PDX models have also proved useful for studies in multiple other cancers, including the prostate, liver, breast, and colon, shown here as a model for cholangiocarcinoma (CC) (Fig. 4C). The representative CC image shown in Figure 4C reflects the complex, multi-cellular tumor with stroma and cells arranged into disorganized structures similar to *de novo* human tumors.

Results from the histotripsy treatments of the subcutaneous RCC, PDX CC, and orthotopic 4T1 mammary tumors showed that histotripsy was able to generate well-defined bubble clouds within each tumor using our small animal system that we clearly visualized on real-time ultrasound imaging (Fig. 4D–F). Using the automated ablation strategy, volumetric ablations of the targeted regions of the tumor were achieved for all three tumor types (Fig. 4G–I). While the central region of the tumor targeted with histotripsy was fully ablated, the external margins of the tumor was still intact after treatment, similar to previous reports [4, 17, 18]. More complete ablations of the entire tumor volume could not be achieved in the small animal models due to the inability to treat sufficient margins around the tumor, particularly on the portion of the tumor located immediately below the skin. When treating near these regions, prefocal cavitation was often noted during treatment at the skin-water interface, limiting the ability to generate a bubble cloud immediately below the skin and causing potential cavitation damage to the skin surface, which was observed in some subjects. With the stiffer CC tumors, the bubble cloud appeared smaller and less dynamic on ultrasound imaging compared to the other tumor types. For this reason, the CC treatments were applied at a higher treatment dose with finer spacing between adjacent treatment points in order to ensure complete ablation of the targeted tumor volume. It was also noted that there was more variability in the appearance of the bubble cloud throughout treatment in heterogeneous tumors, with regions of suppressed cavitation. These areas of suppressed cavitation likely correspond to the more fibrous regions of the tumor. Overall, results showed that histotripsy could be successfully applied to all murine tumor types tested in this study, resulting in complete ablation of the targeted central region of the tumor with the primary limitation being the inability to treat the entire tumor volume and a clinically relevant treatment volume around the tumor. Regardless of the limitations, we have found that the mouse models utilized thus far for histotripsy development, including those shown in this study, are ideal for rapid and reproducible parameter evaluation, hypothesis testing, and effective assessments of biological hallmarks of cancer impacted by treatment. These models can also be used to assess key engineering parameters necessary for device scale-up in certain cases, such as comparing the relative differences in pulsing parameters needed to ablate different tumor types.

### B. Characterization of RAG2/IL2RG Pig Tumor Model

For the current case study, we utilized 454 *in vitro* fertilized oocytes, which were injected with the RNA form of CRISPR/Cas9 targeting *RAG2* and *IL2RG*, followed by *in vitro* culture [96]. Four days post-IVF, 155 embryos between the 8-cell and morula stage were transferred to surrogate gilts. This resulted in 6 piglets being delivered by hysterectomy into our germ-free house system, 114 days post-IVF. Genomic DNA collected from tail-snips revealed that all 6 piglets were successfully targeted for both the *RAG2* and *IL2RG* genes [96]. Further functional verification from peripheral blood specimens, evaluated via flow cytometry, confirmed that the piglets presented with the B-T-NK-SCID phenotype.

**Fig. 5.**
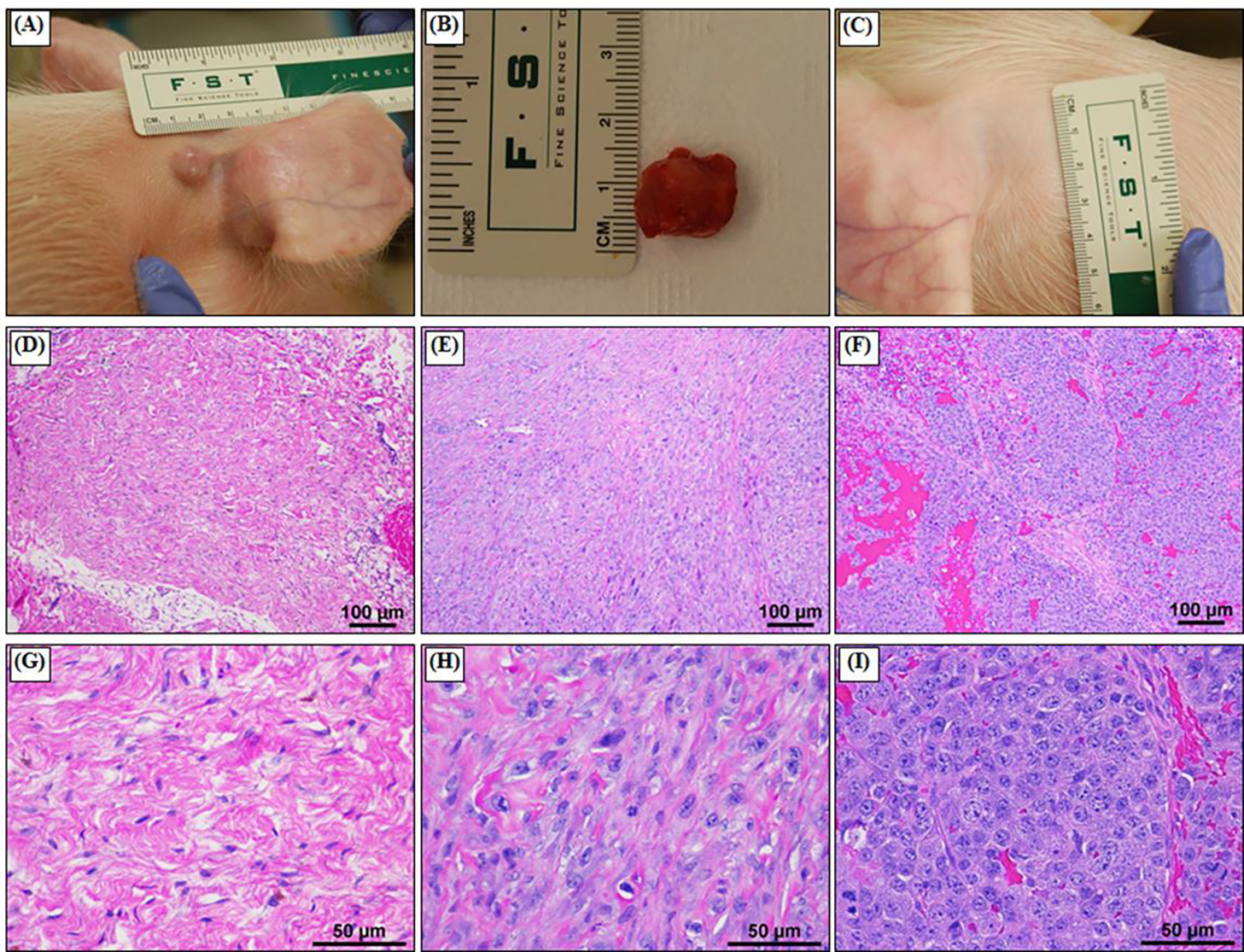
RAG2/IL2RG Immunocompromised Pig Model for Cancer Research. (A) Example of a Pan01 (pancreatic cancer) subcutaneous tumor nodule growing behind the ear and (B) once excised. (C) Control Matrigel plugs exhibits no growth. (D, G) U-251 Human Glioblastoma, (E, H) 4T1 murine breast cancer, and (F, I) HepG2 human HCC are shown by histology under low and high magnification, respectively.

For tumor engraftment, we have evaluated tumor progression of the following human cancer cell lines in the *RAG2/IL2RG* knockout pigs: PANC-1 (pancreatic cancer) (Fig. 5A–B); HepG2 (liver cancer); and U-251 (brain cancer). The murine 4T1 (breast cancer) was also engrafted. These cell lines represent tumor types that we feel could significantly benefit from histotripsy-based applications and are areas of intense research focus in the field. This is the first report of the HepG2, U-251, and 4T1 tumors being successfully engrafted into a porcine model; there is a prior study that utilized the PANC-1 tumors to establish the potential of these animals to support xenografted tumor growth [103]. The 6 piglets were randomized and subcutaneously injected with 1.2×10^6^ cells in 100 μL of Matrigel in the ear. Control injections were also added, which were 100 μL of Matrigel alone, and were not found to produce any tumors (Fig. 5C). Each cell line was injected in triplicate in three separate pigs. In these initial studies, we established engraftment proficiency and growth rates for each cell line. Within 24 hours, of the initial injection, we observed tumor growth for all of the cell lines, except for 1, U-251 injection. This single U-251 injection was the only cell line that failed to engraft. The remaining cell lines rapidly expanded over a 5-day period, eventually stabilizing and steadily increasing to the target diameter of 1.0 – 1.6 cm by day 36. No changes in any clinical parameters were noted for any of the animals. All tumors were readily palpated and observed by day 36, whereas the Matrigel control injections were not detected. Tumors were evaluated by ultrasound, revealing clearly defined tumors and clear delineations from surrounding tissues. Extra-dermal, sub-dermal, and ultrasound tumor measurements were collected at necropsy and compared for each tumor. Metastasis was also evaluated in the lung, liver, spleen, and draining lymph nodes, with none detected. Histopathology assessments were consistent with each tumor cell line, here showing U-251 (Fig. 5D and G), 4T1 (Fig. 5E and H), and HepG2 (Fig. 5F and I) tumors as representative specimens. Each was indistinguishable from the respective cell line-based tumors grown in immunocompromised mice. However, the size of the mice and the number of tumors that can be generated is a significant limitation. This is especially true when transitioning these studies to orthotopic tumor engraftment, where the mouse has significant limitations in terms of human relevance. Future and on-going studies will utilize these data to refine and optimize orthotopic tumor engraftment with these cell lines in the *RAG2/IL2RG* deficient pigs. In addition to cell line tumors, we have also successfully generated PDX-like tissue and tissue organoids from humans and engrafted these more complex and physiologically relevant specimens into the *RAG2/IL2RG* knockout animals, opening the opportunity to move beyond cell line-based tumor models.

### C. Histotripsy Treatment of Porcine Pancreas and Liver

**Fig. 6.**
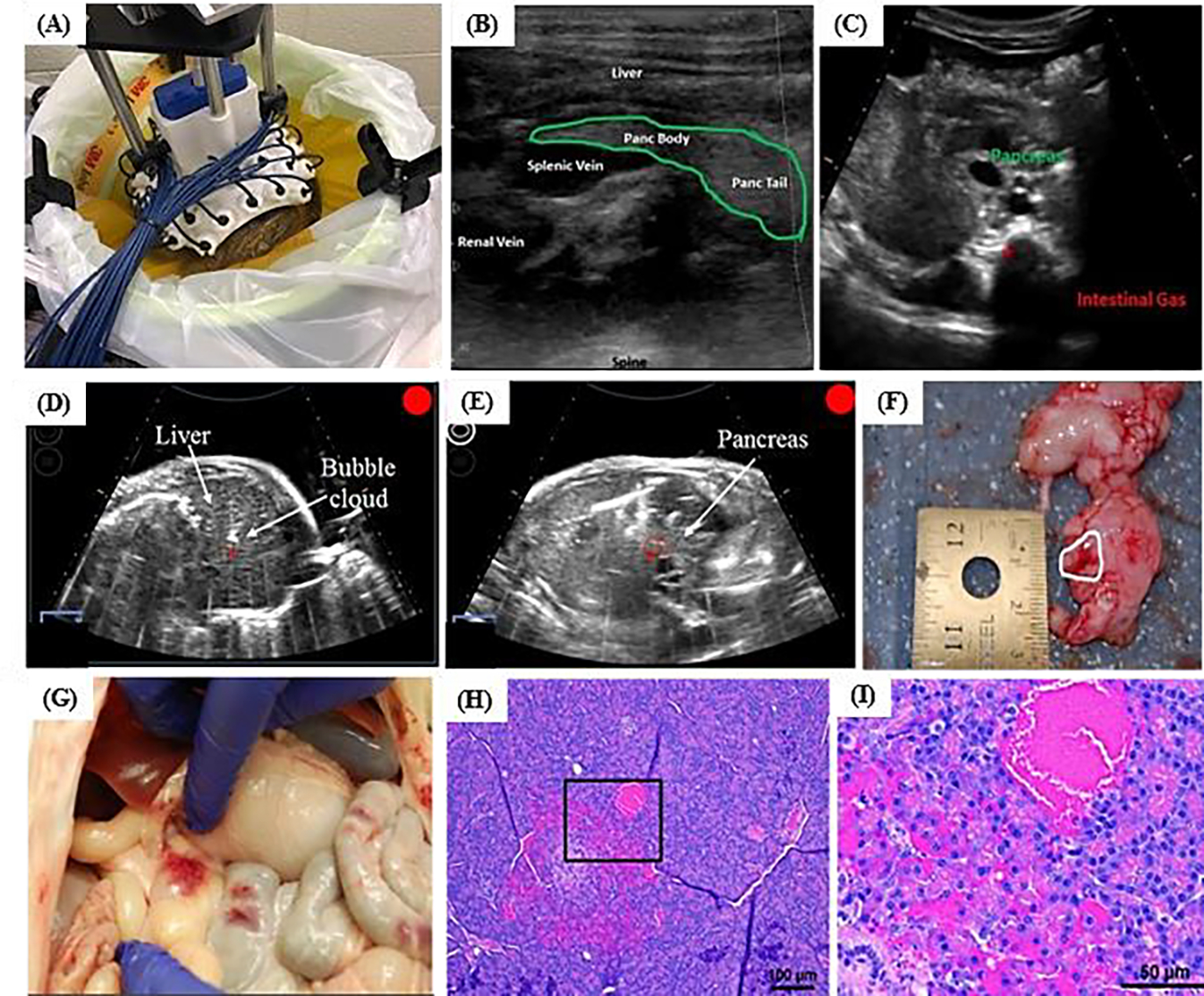
Histotripsy Treatment of Porcine Pancreas and Liver. (A) Image of the water bowl and site preparation with fluid repellent adhesive surgical drape for liquid containment during histotripsy. (B) Acoustic window for pancreas ultrasound treatment identified on pre-treatment imaging. (C) Gas in the stomach and GI tract encountered prior to histotripsy treatment. (D-E) Guided by real-time ultrasound imaging, histotripsy generated a clearly defined bubble cloud in (D) liver and (E) pancreas. (F) Gross image of 0.5 cm lesion in pancreas following histotripsy. (G) Off-target effects around the stomach and GI tract. (H-I) Representative histopathology images confirm regions of ablation in the pancreas.

The feasibility of targeting the liver and pancreas in porcine subjects was tested using the large animal histotripsy device shown in Figure 3. For this feasibility study, treatments were applied non-invasively through an abdominal window and partial ribcage obstruction (Fig. 6A). The liver and pancreas were imaged and targeted in each pig at treatment depths of ~4–6 cm (Fig. 6B–E). In the pancreas, gas in the stomach and gastrointestinal (GI) tract was a significant issue (Fig. 6C). For liver histotripsy treatments, well-defined bubble clouds were generated at the focus of the transducer and clearly visualized as dynamically changing hyperechoic regions at the focus of the transducer, as monitored by real-time ultrasound imaging (Fig. 6D). These results were consistent with previous *in vivo* hepatic histotripsy treatments that have been reported in pig models [1, 3, 11, 12]. In contrast to the results seen in liver, targeting the pancreas with histotripsy posed a more difficult challenge using our current large animal system. Prior to treatment, the pancreas could be identified and visualized on ultrasound imaging when using a freehand ultrasound imaging probe with significant mechanical force applied to the abdomen to displace bowel gas (Fig. 6B–C). However, the quality of the image was reduced significantly when the pancreas was imaged by the co-axially aligned imaging probe within the therapy transducer (Fig. 6E), likely due to blockage from bowel gas (mechanical force could not be applied in this case due to the water standoff). In all subjects, the offset placement of the histotripsy transducer and imaging probe through the degassed water coupling bowl prevented the pancreas from being clearly visualized during treatment. Based on this limitation, the focus of the histotripsy transducer was aligned to the region expected to contain the pancreas using nearby anatomical landmarks, and histotripsy was then applied to this as described in the Methods. During treatment, histotripsy cavitation could be heard, but the bubble cloud was only sporadically visualized in the focal region during treatment (Fig. 6E). In addition, prefocal cavitation was sporadically observed during treatment in regions outside of the focus, likely corresponding to regions containing gas-filled overlying tissues. After treatment, gross morphology showed a small histotripsy lesion in the pancreas, as indicated by a region of localized hemorrhage approximately 0.5 cm in size, smaller than the planned treatment volume diameter of 1 cm (Fig. 6F). In addition to the damage to the pancreas, off target injury to surrounding organs, including the small intestine and stomach, was observed (Fig. 6G), likely corresponding to the regions of prefocal cavitation observed during treatment. Ablation in the pancreas was confirmed by histopathology as a small region of localized tissue damage inside of the pancreas (Fig. 6H–I).

### D. Histotripsy Ablation of Spontaneous Canine Tumors

**Fig. 7.**
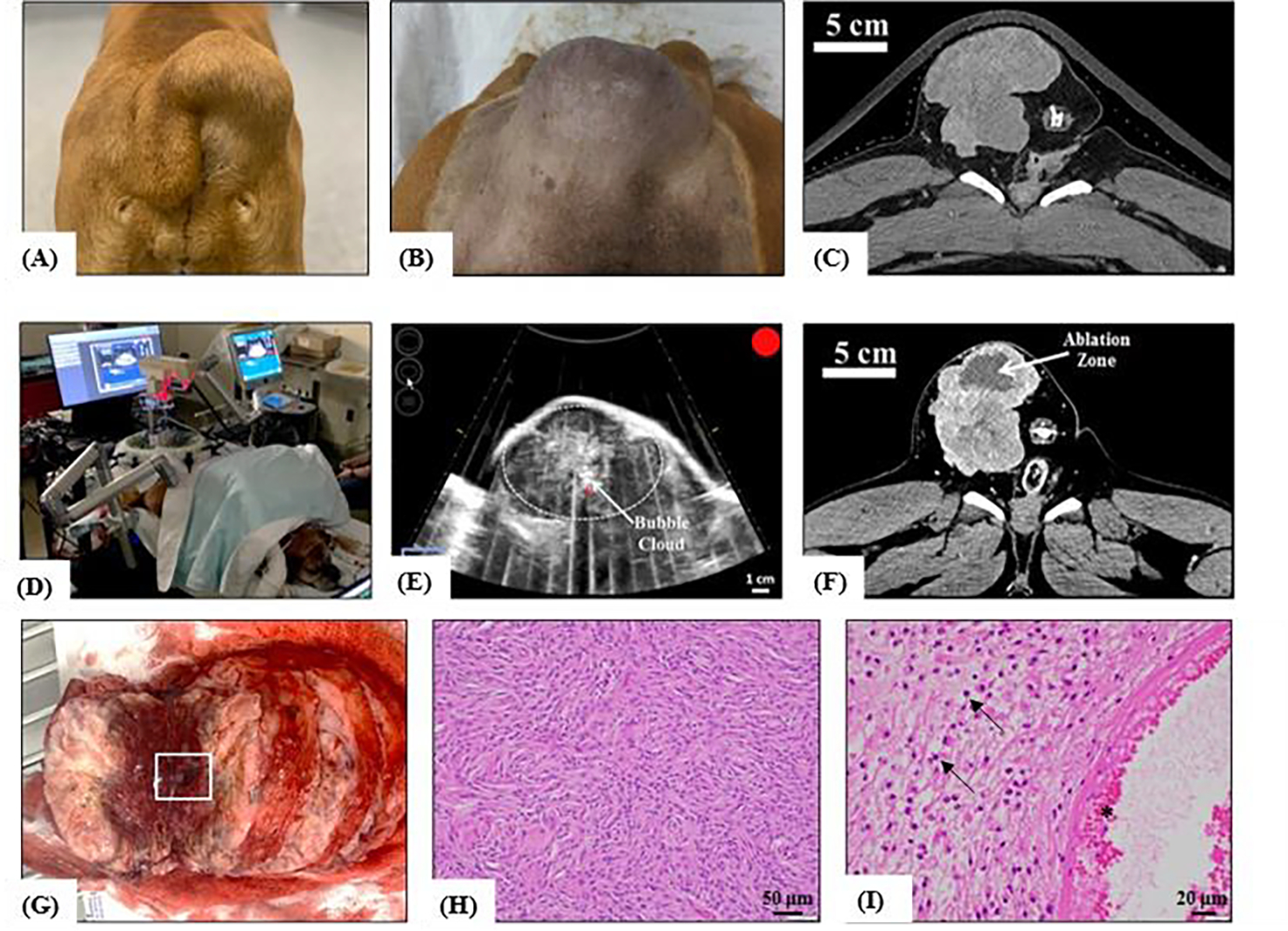
Histotripsy Treatment of Canine Soft Tissue Sarcoma. (A-B) Image of spontaneous soft tissue sarcoma with estimated tumor size of ~7–8 cm in diameter. (C) Contrast-enhanced CT collected before histotripsy treatment. (D) Histotripsy treatment experimental set-up. (E) Real-time ultrasound image collected during histotripsy treatment showing the bubble cloud visible as a hyperechoic, oscillating zone (arrow) contained within the tumor volume (circled). (F) Contrast-enhanced CT collected 4 days after histotripsy treatment with decreased contrast uptake visible in the zone of ablation. (G) Gross morphological analysis of resected canine soft tissue sarcoma. Box indicates treatment region, identified by location and presence of tissue necrosis and hemorrhage. (H) H&E image of untreated canine soft tissue sarcoma (20x magnification) with intact cells and undisturbed extracellular matrix is observable. (I) H&E image of canine soft tissue sarcoma tissue treated with histotripsy (40x magnification) showing extensive cell necrosis (arrows) and hemorrhagic appearance (asterisk).

**Fig. 8.**
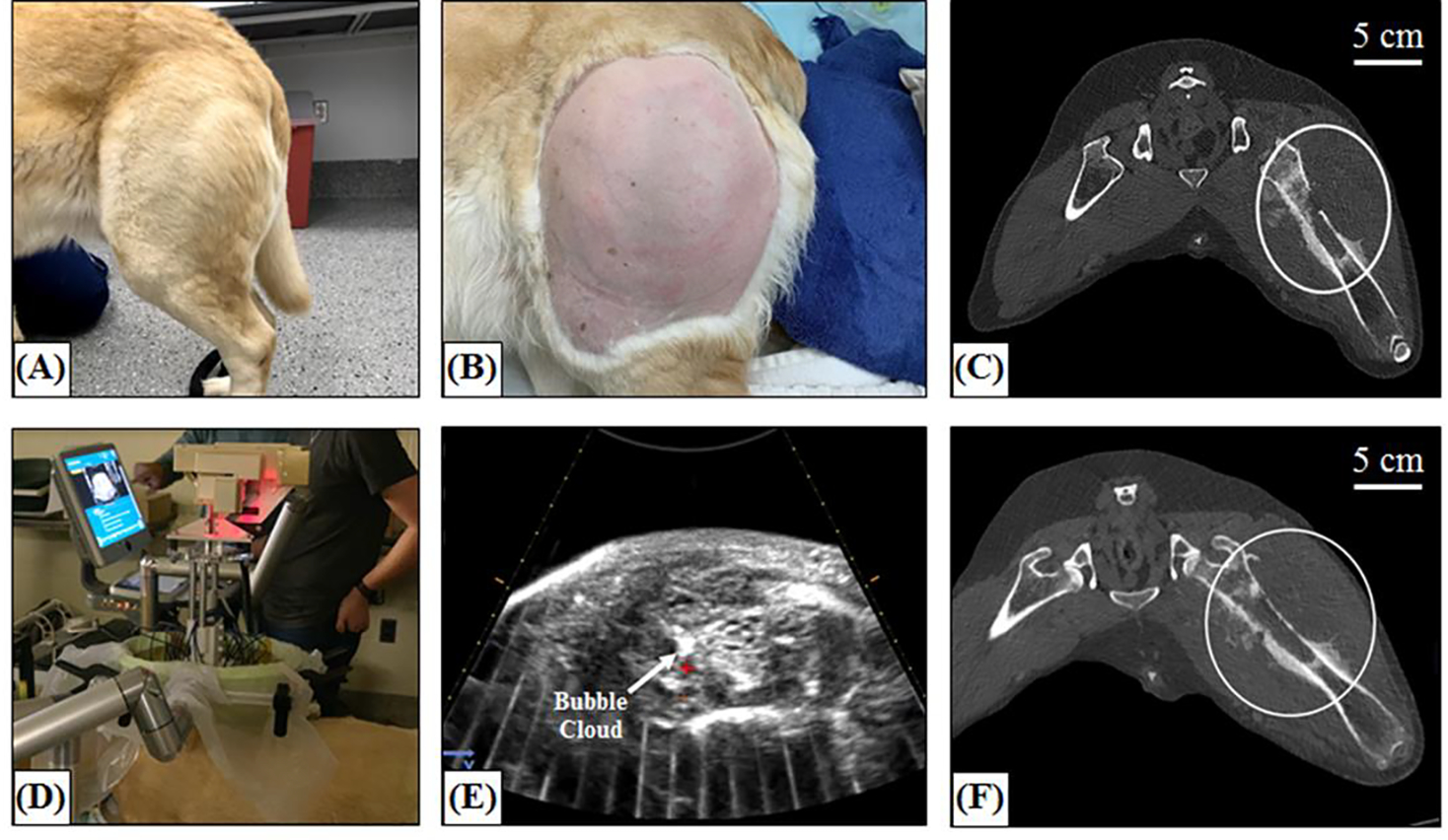
Histotripsy Treatment of Canine Osteosarcoma. (A-B) Image of an undisturbed spontaneous canine osteosarcoma in the left hind limb. Significant soft tissue swelling was present, creating an estimated zone of ~30 cm of affected tissue. (C) Contrast-enhanced CT collected before histotripsy treatment with the region affected by the tumor circled. (D) Histotripsy treatment experimental set-up. (E) Real-time ultrasound image collected during histotripsy treatment with the bubble cloud visible as a hyperechoic, oscillating zone (arrow). (F) Contrast-enhanced CT collected 1 day after histotripsy treatment showing no visible histotripsy ablation zone and the affected region circled.

The feasibility of conducting *in vivo* histotripsy treatments in canine patients with spontaneous tumors was tested in two initial patients with a peripheral soft tissue sarcoma (Fig. 7) and a hindlimb osteosarcoma tumor (Fig. 8). Histotripsy was applied non-invasively at a *p*− estimated to be ~30–35 MPa. A 3 cm spherical ablation volume was targeted inside of both tumors, and histotripsy was applied uniformly over this volume at a PRF of 500 Hz and a dose of 500 pulses/point, with a total treatment time of ~59 minutes. In both dogs, well-defined histotripsy bubble clouds were generated at the focus of the transducer and clearly visualized as dynamically changing hyperechoic regions throughout the treatment, as monitored by real-time ultrasound imaging (Fig. 7E, Fig. 8E). The procedures in both dogs were tolerated well throughout treatment, with no signs of distress or changes in vital signs during the procedures. After treatment, both subjects were recovered until follow-up CT imaging and surgical resection one day (osteosarcoma) or 4 days (soft tissue sarcoma) after treatment. Both the pre- and post-treatment CT images (Fig. 8C/F) for the dog with the osteosarcoma tumor did not show a clearly defined tumor or ablation zone after treatment, likely due to the heterogeneous nature of the osteosarcoma tumor, the presence of significant edema, or CT imaging parameters. Future work will aim to improve the CT imaging for these patients or utilize MRI imaging, as needed. The post-treatment CT image for the dog with the peripheral soft tissue sarcoma showed a clearly defined ablation zone closely matching the region targeted with histotripsy (Fig. 7F). The ablation zone can be clearly visualized on CT as a darkened region of the tumor cross-sectional image (Fig. 7F), indicating a decrease in uptake of contrast and blood flow to this region which correlates with the successful histotripsy treatment. After treatment, gross morphology showed a histotripsy lesion was generated in the targeted tumors, and the tissue was processed for histological analysis after resection. Figure 7G–I shows an example of gross morphology and histology for the peripheral soft tissue sarcoma tumor treated with histotripsy, with results showing histotripsy generated complete ablation of the targeted tumor tissue. Together, results from these initial treatments provide an example of histotripsy tumor ablation in dogs with spontaneous tumors and demonstrate the potential of utilizing veterinary oncology populations for conducting more clinically relevant studies of histotripsy for cancer applications.

## IV. DISCUSSION

While early diagnosis and multi-scale combinatorial treatment approaches are essential to reducing cancer related mortality, the lack of treatment options for many types of cancers has resulted in an unacceptable stagnation in patient morbidity and survival. New treatment options and therapeutic strategies are critical to improve the survival of these patients. Focal tumor ablation modalities have shown recent success in multiple clinical trials by crossing barriers that are difficult to bridge by chemotherapy and surgery. Histotripsy is emerging as one of the more promising modalities. However, as with all of the other tumor ablation strategies, clinical translation for many of the most difficult to treat malignancies has been hampered by the lack of robust pre-clinical animal models. While some modalities, such as radiotherapy and microwave ablation, have the historical advantage of multiple years of clinical data in human patients, it is much more difficult for emerging technologies, such as histotripsy, to be gain widespread adoption in the human clinic without more mechanistic insight that defines their therapeutic advantages. Indeed, the non-thermal nature of these modalities appears to have significant benefits and increasingly nuanced mechanisms that require more robust assessments to fully understand their mechanisms of action. For example, it is becoming increasingly apparent that these modalities are significantly better than currently adopted strategies at engaging the immune system following treatment [17, 33]. This has significant implications associated with the so-called “abscopal effect” [104], which appears to occur more predictably and robustly following non-thermal ablation. This has multiple benefits, including reduced metastatic burden, lower recurrence rates, improved responsiveness to immunotherapeutics, and improved overall survival. However, the improved systemic effects associated with local tumor ablation significantly increases the complexity of the studies necessary to validate these mechanisms, thus, necessitating the development of improved pre-clinical animal models for both acute and chronic assessment [2, 4, 17, 28, 105].

In terms of animal models, the use of rodents in tumor ablation studies continues to be widely accepted. This is certainly understandable due to the historic use of mice and rats in the cancer biology and biomedical engineering fields. Indeed, we have already mentioned several advantages and disadvantages, such as the small size that can be highly detrimental for engineering and device design. This often necessitates the miniaturization of devices that work well in rodents but either fail to scale up and translate well to humans or require additional validation in large animal studies prior to regulatory approval for human testing. For example, the histotripsy system used to treat mouse tumors in this study consisted of a highly customized device that can only target small, superficial tumors at depths <1.5 cm from the skin. While the smaller bubble cloud generated by this system allows for more precise treatments and finer control over ablation margins in the murine model, using this system to treat tumors in mice does not answer key questions about the potential feasibility of treating tumors in human patients through the acoustic windows available for a given pathology. It also makes it difficult to determine the optimal approaches for achieving complete volumetric ablation of human tumors (with adequate clinical margins) that are located in different regions and with a wide range of characteristics including tumor size, the number of nodules, and the presence of critical structures within or near the tumor. In addition to the limitations of the therapy transducer, the small animal system also utilizes a high frequency ultrasound imaging system that provides excellent real-time treatment guidance and monitoring but only provides minimal information about the potential to guide and monitor histotripsy treatments in future clinical studies, which will require a lower frequency ultrasound imaging probe capable of imaging increased depths and a wider field of view. Although small animal tumor studies are often supplemented with experiments in healthy large animals, histotripsy ablation of healthy tissue does not allow for investigations into whether histotripsy can achieve precise and complete ablation of a tumor nor the ability to assess real-time and post-treatment imaging methods to separately visualize the ablation zone from residual untreated tumor. Finally, these limitations make it difficult to assess the safety of histotripsy for treating larger tumors that are typically seen in humans as well as the safety of ablating tumors in an animal with similar anatomy and physiology to human patients.

In many ways, the failure to translate new ablation methods, such as histotripsy, into the clinic is associated with the choice of cancer models used to evaluate, validate, and optimize therapeutic strategies. For example, most tumor ablation studies have taken advantage of subcutaneous tumor engraftment models, especially during early modality development. As discussed in our results section, these models have several advantages and are highly useful, especially during early proof-of-concept/proof-of-principle testing. The subcutaneous engraftment of human cancer cell lines in immunocompromised mice, for example in the flank, have a long history of use in studying cancer hallmarks. In terms of histotripsy, these models offer a readily accessible tumor where the direct effects of ablation can be evaluated, both visually and by ultrasound. Likewise, the use of human cells in the mouse allows for straightforward visualization of ablation using human and mouse-specific antibodies and other reagents to label the human-derived tumor cells and host specific mouse cells. Unfortunately, as shown and discussed in our results, tumors generated subcutaneously and using cell lines have several disadvantages. Specifically, the tumor morphology, biology, and stroma are significantly altered from the original host tumor. In the context of histotripsy, this is a significant concern where tissue characteristics, particularly tissue fibrosis and other factors that impact the local tissue mechanical strength, can significantly impact ablation success [6, 9, 14, 106, 107]. Without adequate models that can recapitulate the relevant tumor microenvironment, it is difficult to identify the optimal histotripsy parameters and treatment doses needed to achieve complete tumor ablation or to develop improved histotripsy strategies for developing tumor-selective ablation methods that can ablate the tumors while preserving critical structures such as vessels, nerves, and bile ducts [1, 3, 9, 13, 14]. Likewise, the lack of a functional immune system does not allow the evaluation of this increasingly important aspect of the tumor microenvironment that may have significant implications in histotripsy effectiveness.

The use of rodent derived cancer cells in the host species is a significant improvement over the use of human cell lines in immunocompromised mice. These cell lines are more amenable to orthotopic studies, where the cells can be engrafted into the tissue of relevance in immunocompetent mice. This provides significantly increased relevance, a more accurate host tissue stroma, and the intact immune system allows for assessments of both local and systemic anti-tumor responses following treatment, which is becoming more essential based on recent studies showing the potential immunological benefits of histotripsy. The ability to genetically modify both the host and the cancer cell line also allows for robust mechanistic studies. It should be noted that the mouse strain and sex are both critical factors to consider in these models. For engraftment to be successful, the strain (i.e. C567Bl/6 or BALB/c) of the host and the cell line must match or the host immune system will reject the tumor. Likewise, male derived tumor cells can fail to engraft in female hosts due to Histocompatibility Y antigen incompatibility and tissue rejection. Thus, while the intact immune system is an advantage, this can limit tumor models in rodents. Likewise, similar to the limitations discussed above for human cell lines and as shown in our results here, the same limitations associated with unrepresentative tumor microenvironments, lack of cell diversity within the tumor, and inaccurate tumor-associated stroma all exist in mouse cancer cell line derived tumors. Each of these are major issues for histotripsy development.

To circumvent many of these limitations, our research team has recently deployed PDX models for histotripsy development [32]. PDX rodent models utilize the engraftment of cancer tissue, typically biopsy or surgically resected tissue fragments, into immunocompromised mice. These mice are typically NOD scid gamma (NSG) mice. As shown in our results section, over time these tumor fragments will proliferate into a tumor that closely mimics the biological and stromal complexity of the original patient’s tumor. The tumor can be expanded to provide ample tissue for *ex vivo*/*in vitro* studies necessary for device development, the tissue can also be evaluated in the animal. Likewise, the added ability to detect human cells in mice can provide high resolution assessments of tumor ablation. While this method still suffers from the lack of an immune system niche, this strategy is incredibly useful for the evaluation of tumor specific ablation to define parameters that are impacted by unique tumor microenvironments, cell complexity, and stroma characteristics.

Moving beyond mice, large mammal pre-clinical models can also be effectively incorporated into tumor modality development. Here, we propose the incorporation of pigs due to their overall anatomical and physiological similarities to humans. Likewise, as we discuss in the results above, genetic manipulation is now becoming more commonplace and the pig is emerging as a key cancer model due to the development of unique animals, such as the immunocompromised *RAG2/IL2RG* deficient pigs that are receptive to cancer cell engraftment, and the rise of genetically modified animals, such as the “oncopig” [40]. In terms of histotripsy development, we have focused on the *RAG2/IL2RG* deficient pigs. These animals are functionally similar to the NSG mice, mentioned above, and we have found that they can be used in identical applications. Advantages of this model include the ability to surgically implant human (or other species) cancer cell lines in specific locations within organs of interest, such as adjacent to major blood vessels or nerves. The cell lines have a predictable growth rate when implanted either subcutaneously or *in situ*, allowing for a highly reproducible and effective model system to evaluate tumor ablation modalities. It should be noted that the use of the CRISPR/Cas9 system allows for the generation of animals “on-demand”, without the need for maintaining breeding colonies. However, one disadvantage is that the targeting of the *RAG2/IL2RG* genes are less predictable. For example, as mentioned in the results section, one of the U-251 cell lines did not successfully engraft in the pigs. Further genotyping of this animal revealed only a partial disruption of the *RAG2* and *IL2RG* genes, resulting in an attenuated but still viable immune system that drove tumor rejection. Thus, before orthotopic injection, it is critical to fully determine both genotype and immune system phenotype in these animals. It is also important to note that due to their immunocompromised status, these animals require housing under germfree conditions that are only available at a small number of institutions worldwide. Moving beyond human cell line studies in this unique model, which have some of the same limitations discussed above for the mouse studies, these animals are uniquely amenable to more complex human tissue engraftments, such as from PDX tissues and organoids. Thus, as we move into the future, these highly useful specimens will allow for orthotopic engraftment in these unique animals for more robust histotripsy development, refinement, and treatment optimization.

We have successfully incorporated porcine models into our tumor ablation modality testing pipeline. In addition to the tumor models discussed above, healthy pig models have already proven useful in defining hepatic histotripsy treatment parameters utilized in current and planned clinical trials [1, 16]. By expanding these studies to include liver tumors in the xenograft pig models, we expect to take advantage of the features of healthy pig models that are anatomically similar to humans. This model will also allow for studying tumor-specific therapies and imaging methods that cannot be tested in healthy animals, such as the safety and effectiveness of ablating liver tumors located near critical structures or the ability to visualize and target liver tumors in locations that require acoustic access through partial or complete rib coverage.

In addition to the liver, the work in this study has started to extend the use of histotripsy as a non-invasive method for ablating pancreatic tumors. The results presented above report the first instance of histotripsy being used to target the pancreas in a large animal model. This is an important milestone, but also illustrates some of the difficulties in treating the pancreas and further supports the use of large animal models, such as the pig, in device development. The acoustic window is critical to most applications of histotripsy, including pancreatic cancer, and it cannot be effectively replicated in mouse models as noted previously. As we report here, gas in the stomach and GI tract can significantly limit the ability to visualize the pancreas during histotripsy procedures and may also limit histotripsy from generating a precise and effective bubble cloud in the pancreas without causing off-target cavitation injury (Fig 6). Protocols to better alleviate these issues are hypothesized to range from engineering changes to the device to refining our imaging and treatment parameters for use in pancreatic cancer, as well as, investigating other changes such as diet adjustments, pharmaceutical interventions, and surgical/interventional strategies to remove excess gas prior to treatment. For example, ongoing work in our lab is investigating the addition of custard to the pigs’ diet and optimized a cocktail of simethicone (anti-gas) and Bisacodyl (laxative) as a less invasive strategy to improve the acoustic window, similar to studies reported for HIFU treatments [108, 109]. Finally, it is possible that alternative targeting methods to ultrasound imaging guided procedures may be needed for targeting pancreatic tumors, such as the use of MRI or CT guided methods that have been used for other focused ultrasound ablation applications in which ultrasound guidance is limited [110–113]. In addition to these improvements, we also anticipate that tumors in the pancreas will be easier to image and target compared to the healthy pancreas, which is an essential benefit of the pig model described in this work. Moving beyond the pancreas, in this report, we describe the use of multiple cell lines from cancers currently being evaluated by our research team to expand on the previously reported pancreatic tumors [103]. In the future, we plan to incorporate histotripsy data generated here for the liver and pancreas to refine the treatment parameters of these other cancers, defined here in subcutaneous models, including the use of PDX specimens and organoids to provide an even more robust and translational model for both human and veterinary patients with a wide range of cancer types.

In addition to human applications, tumor ablation modalities such as histotripsy are also emerging therapeutic strategies in the veterinary clinic, especially in veterinary oncology. Ablation techniques such as histotripsy offer an attractive alternative treatment to surgical resection of tumors. The current standard-of-care for many tumors in veterinary oncology includes surgical resection of the primary tumor. However, surgical resection sometimes is accompanied by excessive risk and morbidity. For example, limb salvage surgery in dogs to resect the primary tumor in OS is associated with a high rate of complications, with major complication rates as high as 71% reported [114]. Additionally, despite the advancements in chemotherapeutics, many veterinary cancer patients, like their human counterparts, eventually succumb to metastatic disease. Histotripsy, with its ability to disintegrate solid tumors and its potential for immunogenic induction of an anti-tumor response, is uniquely poised to achieve the major goals of cancer therapy – treating both the primary tumor and metastatic lesions. It should also be noted that the case study presented here did not use a higher dose for the osteosarcoma treatment given that it was subcutaneous and less calcified. However, we expect that a higher dose may be needed for osteosarcoma tumors inside bone in the future, at magnitudes similar to the cholangiocarcinoma tumors.

Not only can veterinary cancer patients directly benefit from novel ablation technologies such as histotripsy, they also provide excellent clinical trial populations for comparative oncology studies to benefit humans. The anatomic, physiologic, and cancer biology similarities between many of the animal species treated in the veterinary clinic to human cancer patients offer a direct translation of technologies from bench-to-kennel-to bedside [115]. For example, the results from the histotripsy treatments of canine STS and OS shown in this study highlight many of the benefits of testing histotripsy in dogs with spontaneous tumors. More specifically, treatments were conducted with a clinical prototype histotripsy system that consists of the therapy transducer, imaging system, and volumetric ablation treatment strategies that are similar to those expected to be used in humans. The results from these studies, which demonstrated histotripsy could achieve precise and complete ablation of targeted volumes within both the STS and OS tumors, are expected to be more directly translatable to humans when it comes to the devices and treatment parameters used for histotripsy. The STS treatment, specifically, showed a clearly visible region of damaged tissue on a post-treatment CT image that matched the region targeted with histotripsy. The necrotic tissue zone was slightly larger on the CT image than the targeted volume. Future work will investigate this phenomenon, but authors hypothesize that the CT image may show not only the region homogenized by the histotripsy treatment, but also a larger region of non-viable tumor tissue. It is expected that this observation may translate to human histotripsy treatments as well.

In addition to providing a more clinically relevant population for testing the safety and efficacy of histotripsy devices, the dog as a comparative oncology research model also offers the unique advantage of being an outbred species with an intact immune system that develops spontaneously occurring tumors over an extended time, just as humans do, unlike inbred laboratory rodents that are not syngeneic host-tumor models. While the current case studies focus on safety and efficacy, future studies utilizing canine models of spontaneous disease allows for evaluation of immune responses to treatments, an evaluation that is not as readily achieved in immunodeficient rodent models. Dogs and humans are more alike anatomically and physiologically than are mice to humans and share the same physical environments, unlike laboratory rodents maintained in a highly controlled research facility environment [59]. Dogs with spontaneously occurring tumors represent powerful tools for device development and clinical trials, as they are a biologically relevant disease model that can serve to better bridge preclinical murine study findings with human clinical trials. Additionally, the willingness of canine owners to engage in sample collections and procedures such as biopsies make dogs an invaluable model for clinical testing. Conversely, client-owned dogs present limitations such as the low prevalence of certain tumor types, for example, intestinal cancer, compared to humans, thus limiting the study of these diseases.

Although only two example histotripsy systems were used in this study for small and large animal experiments, respectively, the results from these studies provide a representative example of the potential histotripsy systems and procedures that can be tested in the various animal models. For instance, the histotripsy system used for the large animal treatments offers several advantages over the small animal histotripsy system when considering the extent to which the results are directly translatable to clinical treatments. Unlike the small animal histotripsy system, which can only target tissues at a maximum depth of ~1.5 cm under the skin’s surface, the working distance of the large animal histotripsy system makes it possible to treat tissue depths up to ~7 cm. This increased treatment depth allows for treatments of the pancreas and liver and a wide range of tumor types in canine including superficial musculoskeletal tumors and abdominal cancers. Like the small animal system, the large animal device uses real-time ultrasound imaging to guide and monitor the histotripsy treatments. The large animal device, however, provides lower resolution imaging but more clinically relevant assessments of image guidance for histotripsy devices that are being developed for human applications by utilizing lower frequency imaging probes to image at clinically relevant treatment depths in the pig and dog models that closely match human anatomy. Additionally, it is worth noting that the larger bubble cloud generated by the large animal histotripsy device allows for more rapid ablation of larger treatment volumes using histotripsy while maintaining sufficient treatment precision for targeting a predetermined volume with the target tissue. This finding highlights how the large animal studies can be utilized for testing of clinically relevant devices and treatment methods to optimize experimental protocols prior to clinical use. For instance, recent studies have developed improved robotic guidance systems that can only be properly tested in large animals that replicate human anatomy [116–118]. Similarly, studies investigating electronic steering for rapid volumetric ablation require large histotripsy array transducers that will likewise need to be tested in large animal models in order to establish *in vivo* efficacy prior to clinical translation.

## V. CONCLUSION

This paper highlights a diverse range of small and large animal tumor models being developed for investigating histotripsy’s oncological applications. Results of example histotripsy treatments performed in mouse models demonstrate the feasibility of using mice as highly cost-effective models that can test histotripsy for a nearly limitless range of cancer types; however, results also show the limitations of mouse tumor models due to their small size and the inability to test clinically relevant histotripsy devices. To address these limitations, we utilized CRISPR/Cas9 RAG2/IL2RG deletion pigs and healthy pigs to demonstrate the strengths of these animals as a model for human-scale devices. Finally, this work highlights the potential benefits of developing histotripsy for the treatment of spontaneous tumors in veterinary patients and utilizing these populations as more realistic models for developing and testing histotripsy devices and techniques for use in human patients. Together, these studies demonstrate the potential advantages of various animal models in the development of histotripsy for oncological applications.

## Figures and Tables

**TABLE 1:** Characteristics of the Small and Large Animal Histotripsy Systems.

Histotripsy System	Frequency & # Elements	Depth to Focus	Aperture Size		F-number		Full Width Half-Maximum (FWHM)	
Small Animal	1 MHz, 8 elements	36 mm	52.7 mm		0.68		*Transverse*	0.98 mm
							*Elevational*	0.93 mm
							*Axial*	3.9 mm
Large Animal	500 kHz, 32 elements	78 mm	*Transverse*	128 mm	*Transverse*	0.61	*Transverse*	2.1 mm
							*Elevational*	2.1 mm
			*Elevational*	112 mm	*Elevational*	0.70		
							*Axial*	6.6 mm

The differences between histotripsy systems used for the small animal and large animal are highlighted using a breakdown of their critical characteristics (frequency, number of elements, aperture, f-number, FWHM, bubble cloud size).
